# Supplementary material for: Functional impact of PCSK9 variants on LDL uptake in a knockout hepatic model
Source: PLoS One. 2026 Oct 26;21(10):e0345908. doi: 10.1371/journal.pone.0345908 (PMC13704872; doi:10.1371/journal.pone.0345908)
Supplement: Supplementary file 1 [file pone.0345908.s001.pdf]

Original, unedited images of blots in Figure 2B

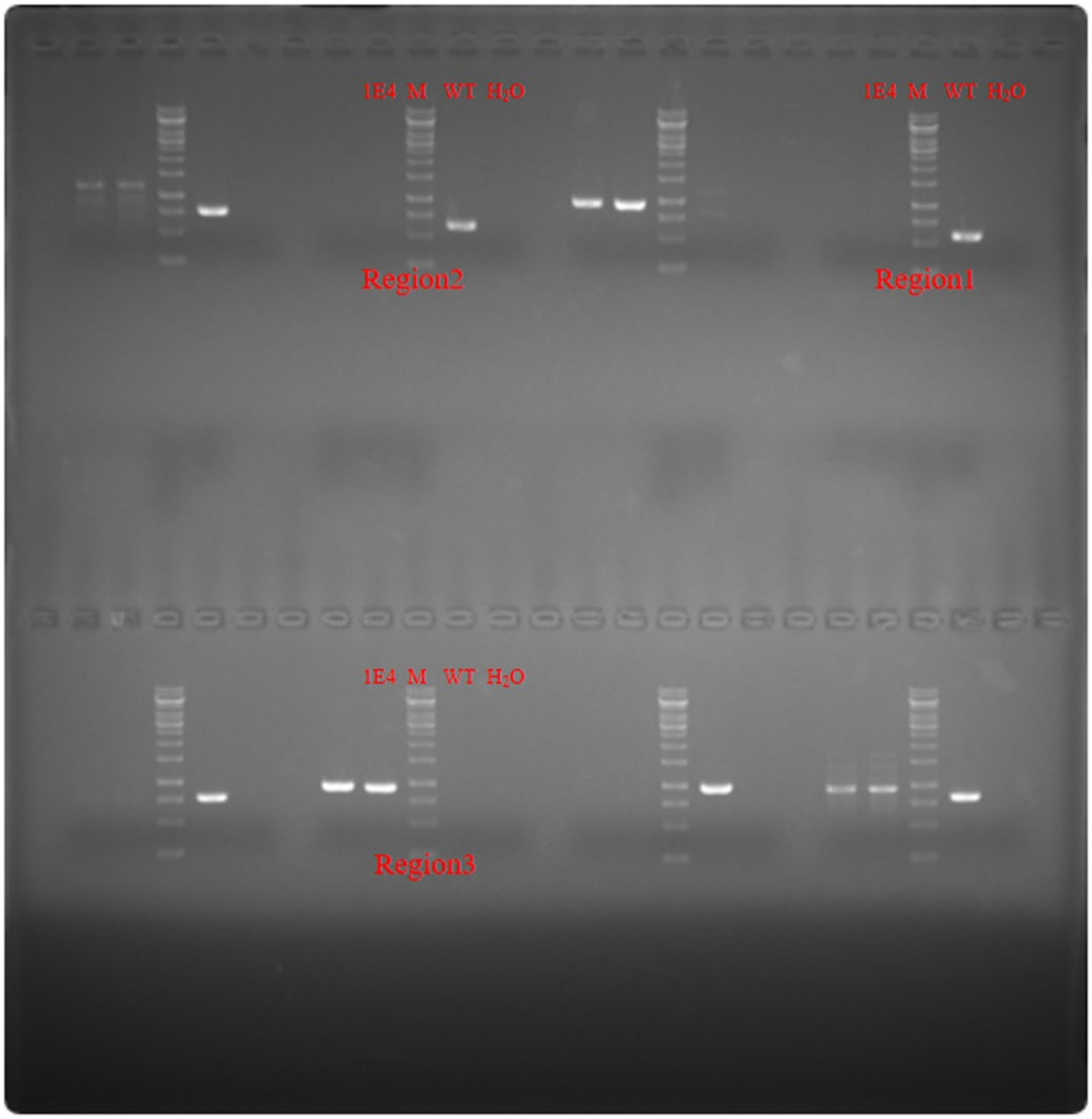

PCR results for Clone 1E4. Red labels indicate PCR fragments corresponding to regions 1, 2, and 3. Unlabeled bands in the gel image represent PCR products from unrelated projects.
