# Supplementary material for: Functional impact of PCSK9 variants on LDL uptake in a knockout hepatic model
Source: PLoS One. 2026 Oct 26;21(10):e0345908. doi: 10.1371/journal.pone.0345908 (PMC13704872; doi:10.1371/journal.pone.0345908)

Original, unedited images of blots in Figure 5B

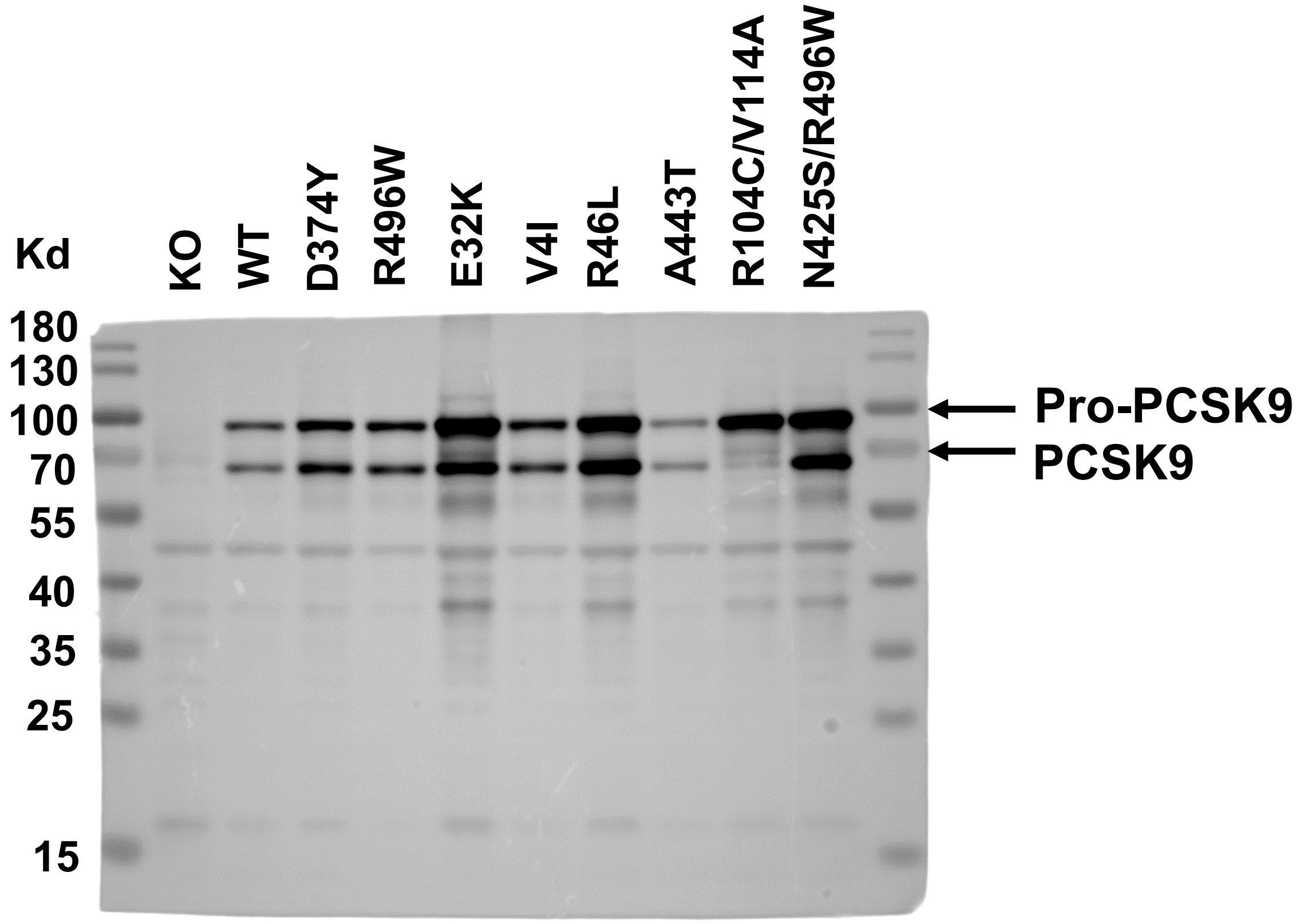

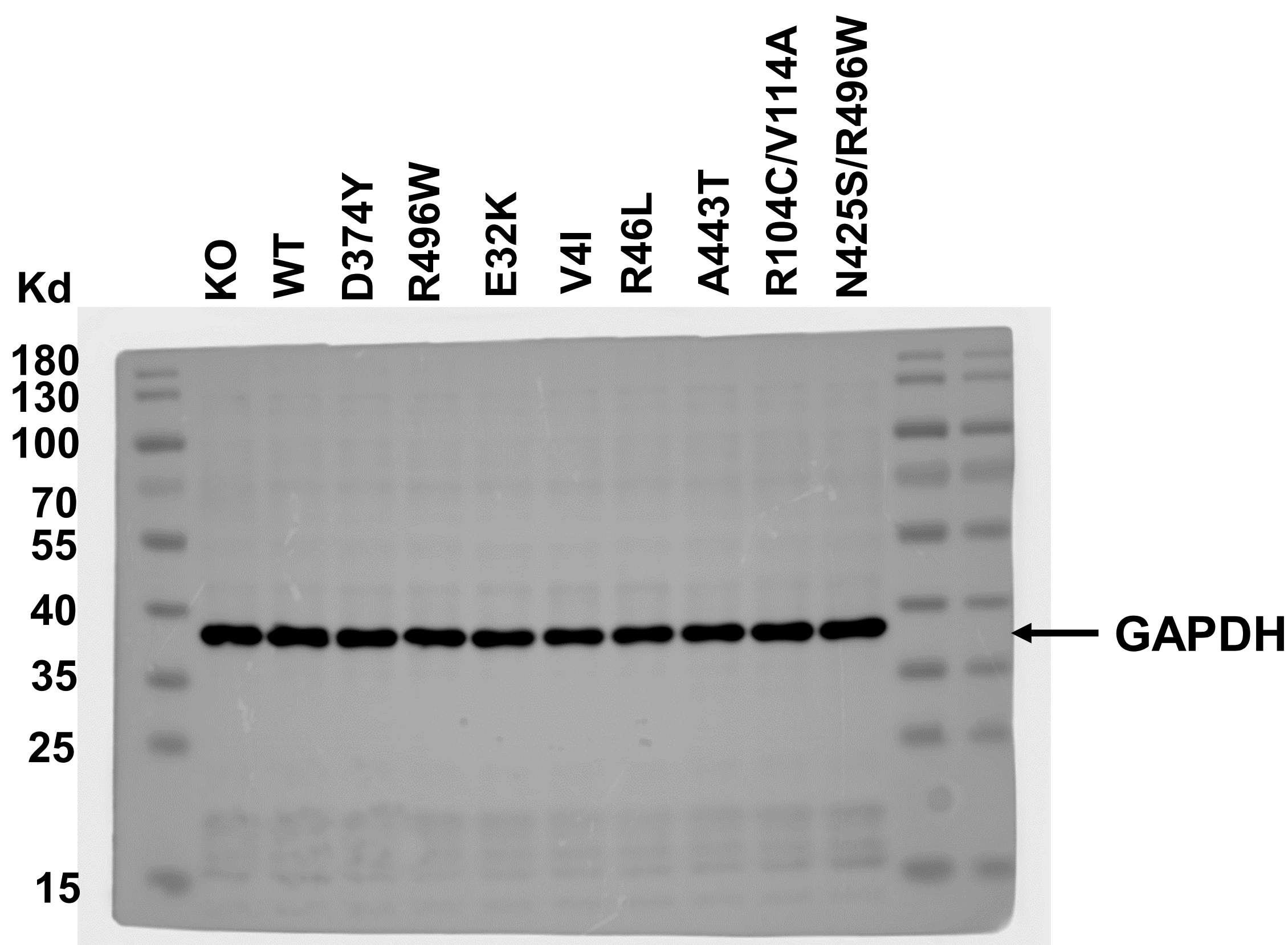

Supplement: Supplementary file 4 [file pone.0345908.s004.pdf]
