# Supplementary material for: Functional impact of PCSK9 variants on LDL uptake in a knockout hepatic model
Source: PLoS One. 2026 Oct 26;21(10):e0345908. doi: 10.1371/journal.pone.0345908 (PMC13704872; doi:10.1371/journal.pone.0345908)
Supplement: Supplementary file 5 [file pone.0345908.s005.pdf]

Figure 4A

Sample	count	Median	Mean
NEG	3122	1325	1620
PCSK9 WT	2872	113139	134353
1G9	2566	142899	165900

Figure 4B

HepG2	1G9
134353	165632
142566	160869
143333	165900

Figure 4C

Neg	HepG2	1G9
2195	13911	14505
	13518	15382
	13150	15410

Figure 5A

1G9 (KO)	WT	V4I	E32K	R46L	D374Y	A443T	R496W	R104C/V114A	N425S/R496W
0.91	55364.234	52136.279	75281.095	82952.624	154795.07	134756.935	144428.907	145769.901	92468.007
1.176	54602.019	52498.915	75804.717	81810.591	134756.935	168221.199	186653.107	144762.995	93111.172
0.935	67222.97	52864.074	74761.091	81810.591	161368.561	165905.248	154795.07	151960.131	100488.206

Figure 5C

1G9 (KO)	WT	V4I	E32K	R46L	D374Y	A443T	R496W	R104C/V114A	N425S/R496W
4.2350375	32.8140882	47.5749497	117.859021	105.4436914	55.5287962	30.147699	46.8103719	66.270613	115.008764
3.9189075	32.8833872	45.2633858	124.1114914	102.0807026	60.8976341	30.138782	49.0465283	59.441596	107.553041
3.5020941	30.3690709	44.7215261	122.9742867	100.976818	53.1047709	29.87401	40.4728364	59.190401	107.973162

Figure 6A

WT	V4I	E32K	R46L	D374Y	A443T	R496W	R104C/V114A	N425S/R496W
20250	18932	17642	22543	14053	28743	14543	21282	19873
20288	19984	18432	23384	17542	27432	15432	22098	18764
21213	18934	16543	24322	16453	27654	14987	24563	20324

Figure 6B

WT	V4I	E32K	R46L	D374Y	A443T	R496W	R104C/V114A	N425S/R496W
18687	17780	16367	19736	16423	21038	15896.89	19352	18936
17893	18096	16443	19402	15489	20763	16086.14	20947	17364
19032	17334	17394	20163	16303	22120	15928.24	19093	17374
